# Refusal Rates in Different Types of Deceased Donors

**DOI:** 10.1111/hex.70910

**Published:** 2026-10-05

**Authors:** Chiara Lazzeri, Adriano Peris

**Affiliations:** ^1^ Tuscany Regional Transplant Center Florence Italy

Dear Editor,

We read with great interest the paper by Tan et al. [1]. The authors are to be commended for the broader inclusion of the three types of deceased donors: donors after brain death (DBD) and donors after circulatory death (both controlled—cDCD and uncontrolled—uDCD). Their results are supported by the substantial number of participants (*n* = 505) to the survey, which was largely representative in terms of ethnicity, gender, and age (range 18–79 years) [1].

The paper offers several key points for discussion.

First, the rate of refusal for organ donation was approximately 30% in the survey by Tan et al. [1]. Refusal rates are critical for transplant authorities, as refusal represents a primary barrier to organ donation. Tan et al. [1] provided an ‘indirect’ estimate of refusal rates that reflected the opinions of survey participants. Although these estimates were based on survey responses, the cohort was largely representative of the general population. However, refusals are evaluated by transplant authorities during the active organ donation process rather than through direct population‐wide surveys. According to the Global Observatory on Donation and Transplantation, incidence rates of refusal in 2024 were reported by 52 out of 92 countries, ranging from 10% in Uruguay and Portugal to 90% in Bangladesh [2]. European data regarding refusal rates in deceased donor pathways remain scarce [3]; however, the Italian National Transplant Center reported stable organ donation refusal rates between 2024 and 2025 (28.7% and 28.8%, respectively), a value comparable to that reported in the survey by Tan et al. [1].

A second interesting topic, addressed by Tan et al. [1] is the evaluation of refusal rates in each different deceased donor pathway. Deceased donor pathways differ across several key factors. Donation after brain death (DBD) cases occur within intensive care units (ICUs), where families are approached by the transplant coordinator once brain death has been formally declared—typically several days following admission. Conversely, uncontrolled donation after circulatory death (uDCD) cases occur in the emergency department following an out‐of‐hospital cardiac arrest. Under these circumstances, families are approached in a vastly different clinical context and must decide within a strict, limited time frame due to the time‐sensitive nature of the uDCD protocol. Recently, we described our 8‐year experience with the implementation of the uDCD program across the Tuscany Region (2016–2024). Overall, 226 potential uDCD cases were assessed by local transplant coordinators, with a notable increase following the COVID‐19 pandemic (151 vs. 75 cases; 67% vs. 33%). Furthermore, a significant increase in refusal rates was observed post‐pandemic (25% vs. 6%, *p *= 0.004).

Tan et al. [1] highlights the importance of measuring refusal rates across three deceased donor pathways. Continuous monitoring of these rates is pivotal for transplant authorities to identify temporal trends. Willingness to donate is influenced by demographic characteristics (e.g., ethnicity, gender, religion) and pathway‐specific factors. Consequently, authorities can leverage these insights to optimize public campaigns and tailor communication training for healthcare stakeholders.

## Author Contributions

**Chiara Lazzeri** and **Adriano Peris:** conceptualization and writing.

## Funding

The authors have nothing to report.

## Conflicts of Interest

The authors declare no conflicts of interest.

## Data Availability

Data sharing is not applicable to this article as no data sets were generated or analysed during the current study.
